# Multiple Light Coupling and Routing via a Microspherical Resonator Integrated in a T-Shaped Optical Fiber Configuration System

**DOI:** 10.3390/mi9100521

**Published:** 2018-10-15

**Authors:** Georgia Konstantinou, Karolina Milenko, Kyriaki Kosma, Stavros Pissadakis

**Affiliations:** 1 Foundation for Research and Technology-Hellas (FORTH), Institute of Electronic Structure and Laser (IESL), GR-711 10 Heraklion, Greece; georgia.konstantinou@epfl.ch (G.K.); kosma@iesl.forth.gr (K.K.); 2 EPFL, École polytechnique fédérale de Lausanne, CH-1015 Lausanne, Switzerland; 3 Department of Electronic Systems, Norwegian University of Science and Technology, NO-7491 Trondheim, Norway; karolina.milenko@ntnu.no

**Keywords:** microstructured optical fibers, whispering gallery modes, light localization

## Abstract

We demonstrate a three-port, light guiding and routing T-shaped configuration based on the combination of whispering gallery modes (WGMs) and micro-structured optical fibers (MOFs). This system includes a single mode optical fiber taper (SOFT), a slightly tapered MOF and a BaTiO_3_ microsphere for efficient light coupling and routing between these two optical fibers. The BaTiO_3_ glass microsphere is semi-immersed into one of the hollow capillaries of the MOF taper, while the single mode optical fiber taper is placed perpendicularly to the latter and in contact with the equatorial region of the microsphere. Experimental results are presented for different excitation and reading conditions through the WGM microspherical resonator, namely, through single mode optical fiber taper or the MOF. The experimental results indicate that light coupling between the MOF and the single mode optical fiber taper is facilitated at specific wavelengths, supported by the light localization characteristics of the BaTiO_3_ glass microsphere, with spectral Q-factors varying between 4.5 × 10^3^ and 6.1 × 10^3^, depending on the port and parity excitation.

## 1. Introduction

High quality light trapping in dielectric, microspherical cavities can be readily achieved using whispering gallery modes (WGMs) resonation; attracting constant academic and potential industrial interest [1,2]. In the WGM resonation process light is confined at the inner interface of a high refractive index dielectric microcavity, illustrated as closed-loop trajectory where rays circulate through total internal reflection (TIR). Several types of optical geometries and corresponding materials have been employed for demonstrating and implementing WGM resonation in photonic devices. The confinement of light propagation within a closed, spherical symmetry dielectric cavity, leads to a three-fold modal quantization of the permitted localization states of light within the cavity volume [3], denoted with three complementary quantum numbers, per k-vector, being directly dependent upon the optogeometric characteristics of the cavity. The interface mechanism of TIR on the boundary of a curved surface is inherently a low loss light dispersion process, leading to high quality factors Q denoting light localization up to ×10^8^, upon materials, excitation protocols and resonator geometries used [4]. A great number of photonic devices based on WGM resonation have been presented including chemical and biological sensors [2,5], lasers [6], wavelength routers and metrological devices [7].

The efficiency of WGMs excitation and related Q-factors of the supported resonances critically depends upon the material properties of the resonator (surface roughness, Rayleigh scattering etc.), its geometry, real and imaginary refractive index, launching conditions and far field or near field signal collection method [8]. Prisms [9], gratings [10], angle cleaved [11], and tapered optical fibers [12] were used for the WGM evanescent field, light coupling into microspheres, leading to different Q-factors and practical consideration including stability of coupling, simplicity of implementation, integration, and interaction with the ambient environment [13].

In addition to the above, MOFs have also been used for reading or exciting WGM resonation inside microspherical cavities, starting with the work of Francois, et al., where a microsphere was wedged onto the end face of a collapsed core MOF [14]; others have used hollow core optical fibers [15,16]. An alternative and high integration WGM excitation approach was presented by Kosma et al. by placing a microspherical resonator inside the air capillary of a MOF taper, for providing a compact and robust operation [17]; similar implementations were also presented by other groups [18,19]. Generally, the use of the end face of standard, tapered and MOFs has been evolved into an efficient configuration for exciting WGMs in microspheres, offering advantages such as single port operation, good yield in fluorescing signal collection, robustness, and versatility in functionalizing and trapping the micro-spherical cavity.

Herein, we present light coupling and routing between the MOF taper and a standard optical fiber taper (SOFT), through a BaTiO_3_ microsphere semi-immersed inside the capillary of the end face of the MOF taper; where the SOFT is placed perpendicularly to the MOF. This T-shaped light coupling system provides interesting WGMs excitation and collection with all three created fiber ports along with light paths parities. We anticipate that the proposed T-shaped excitation system can lead to numerous devices and applications of WGM resonators for sensing, filtering and frequency stabilization, and can be used in integrated optical devices as an efficient add-drop filter. The use of BaTiO_3_ microspheres constitutes a base for straightforwardly attaining permanent and/or transient photorefractive tuning of the spectral characteristics of the WGMs using pulsed or continuous wave (CW) laser beams at low pump powers. This type of spectral trimming has already been demonstrated for a variety of materials and WGM resonating geometries [20,21,22]. Potentially, the photorefractive tuning of the WGM spectral characteristics can also happen for light excitation (for example using 405 nm or 532 nm CW lasers) through an all silica MOF, where the photorefractivity of the MOF itself will be minimum. An additional reason for using BaTiO_3_ microspheres is the possibility of permanent encapsulation and fixation of the system MOF-BaTiO_3_-SOFT using standard ultraviolet (UV) glues (see Norland UV adhesives, Norland Products, Inc., Cranbury, NJ, USA) with a refractive index lower than silica, without great compromising of the Q-factors of the excited WGMs [23]. Our investigations include the steps followed for realizing the device presented, and its spectral characterization and discussion for different portal excitations and read-outs.

## 2. Experimental Section

**Figure 1:**
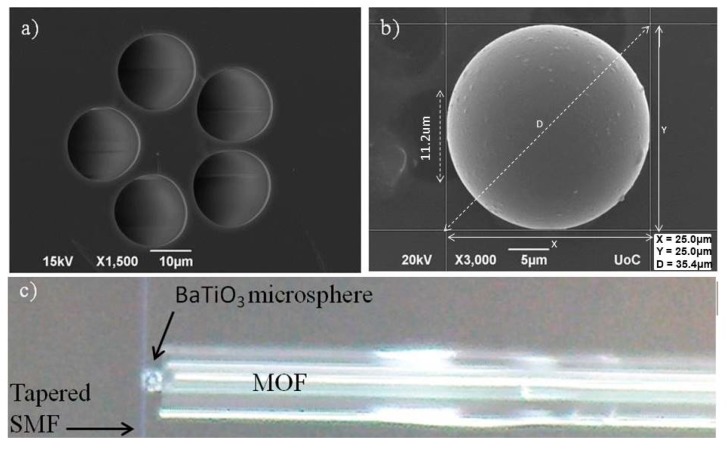
(**a**) Scanning electron microscope (SEM) picture of the un-tapered, grapefruit shaped micro-structured optical fiber (MOF) used, with five air capillaries and germanium doped core, (**b**) SEM image of the attached BaTiO_3_, microsphere with 25 μm diameter, top fitting in one of the capillaries (diameter: 11.2 μm) of the tapered MOF. The other empty capillaries can be seen in the background, (**c**) optical microscope picture of a side view on the T-shaped light coupling system (single mode optical fiber taper (SOFT) placed perpendicular to MOF in a contact with the microsphere).

**Figure 2:**
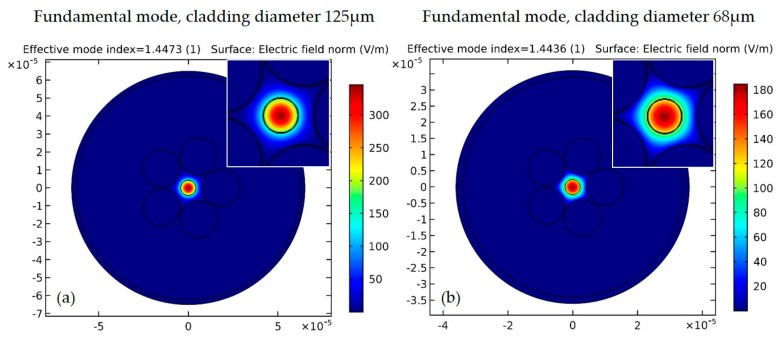
(**a**) Fundamental mode for the MOF in a 2D cross-section image by adopting COMSOL (Multiphysics 3.5, COMSOL Inc., Stockholm, Sweden) simulation. (**b**) Fundamental mode in a 2D cross-section image for a smaller cladding size after the tapering process (68 μm diameter), and accordingly smaller core and air capillaries. Increased spreading of the mode is observed due to reduced spatial confinement.

A grapefruit shaped MOF (drawn by ACREO, Stockholm, Sweden), consisting of two concentric cores, a 3.5% germanium doped silica inner core with diameter 8.5 μm and an outer silica core of diameter 16.1 μm, 20.8 μm diameter 5-air capillaries and 125 μm cladding diameter was used (see Figure 1a). For facilitating efficient wedging of the microspheres available, as well as increasing the evanescence field tail extending outside the microstructured core, this MOF was adiabatically tapered down to ~55% of its initial size and cleaved at the transition region, resulting in a tip diameter of ~68 μm and air capillaries scaling down to a ~11 μm diameter (see Figure 1b,c). For these tapering conditions the fundamental guiding mode is still confined at the Ge doped core of the grapefruit MOF, however, with a more extended modal profile covering the microstructured area. In Figure 2, we show the fundamental guiding mode confinement by using a commercial modal solver.

Additionally, since the adiabaticity criterion was followed, coupling from the central mode to modes supported at the extended microstructured core area is rather limited; the last is confirmed by the absence of significant beating features in the transmission spectra of the tapered MOF. 

Barium titanate (BaTiO_3_) glass microspheres of poly-disperse sizes and typical eccentricities of the order of 10% (Mo-Sci Corporation) were used. The refractive index of these microspheres varies between 1.9 and 2.1, depending on the stoichiometric ratio between Ba and Ti. A BaTiO_3_ microsphere with a diameter 25 μm was attached to the end face of the MOF taper tip while being semi-immersed inside one of its air capillaries (Figure 1b). Air suction was used to ensure stable positioning and attachment of the microsphere. Figure 1 presents scanning electron microscopy (SEM) images of the employed MOF and the BaTiO_3_ microsphere. Additionally, a single mode optical fiber (SMF-28, Corning Inc., Corning, NY, USA) was tapered adiabatically down to 0.9 μm final waist diameter to achieve a broad evanescent field. All optical fibers used were thermally tapered with the use of a Vytran GPX-3000 (Thorlabs, Inc., Newton, NY, USA) optical fiber processing equipment.

**Figure 3:**
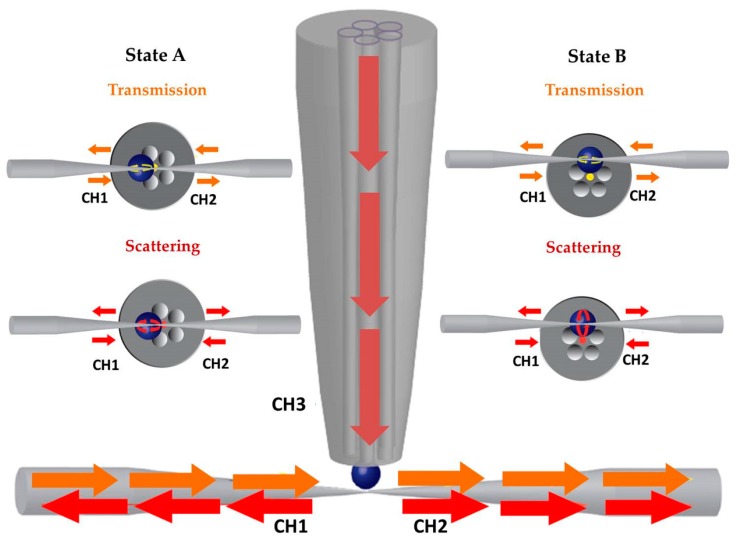
Schematic representation of the experimental setup showing the T-shape excitation system. CH1, CH2 and CH3 represent ports used to excite and measure WGMs. Two possible WGMs excitation configurations are demonstrated in the cross section of MOF taper tip (yellow and red arrows). The yellow arrows are referred to the transmission spectrum (CH1 to CH2 and vice versa) whereas the red arrows are chosen for the scattering spectrum (CH1/CH2 to CH3 and vice versa). Two different states, in terms of orientation between the axis of the SOFT with the axis formed by the MOF taper core and the semi-immersed microsphere, were studied and are presented as State A and State B. In State A the aforementioned two axes are parallel to each other whereas in the State B they are perpendicular.

The MOF taper with the attached microsphere was aligned perpendicular to the waist of the tapered fiber, so that the SOFT waist was in a contact with the equatorial region of the BaTiO_3_ microsphere (Figure 3). In this way a three channel device was implemented, with routing point at the BaTiO_3_ microsphere semi-immersed into the end face of the MOF; namely CH1 and CH2 represent the two portal ends of the SOFT and CH3 the MOF port. Moreover, this three-port optical fiber configuration was characterized with respect to the orientation of the line defined by the MOF core and the BaTiO_3_ microsphere with the axis of the SOFT (Figure 3). In the parallel orientation (State A) the MOF central core and the center of the BaTiO_3_ microsphere are in line with the axis of the SOFT, and the k-vectors of light propagating into the MOF core and the circumference of the BaTiO_3_ along the plane of the SOFT excitation rest along the same axis of propagation. In the perpendicular orientation (State B) the SOFT excites the BaTiO_3_ microsphere, however the axis between the MOF central core and the BaTiO_3_ microsphere is positioned at 90° with respect to the axis of the SOFT, thus the k-vectors of the circumferential WGMs without polar components excited do not lay on the same axis with the MOF core. In the B State orientation, the position of the MOF taper core was below and far from the SOFT horizontal plane, eliminating any potential coupling due to lensing and total internal reflection cancellation. Transmission spectra were obtained for both State A and B of this three port, optical T-junction.

All spectral measurements were made using the amplified spontaneous emission (ASE) of an erbium doped fiber amplifier with a spectral range 1518–1580 nm, whereas an optical spectrum analyzer (OSA) with a maximum spectral resolution of 0.01 nm was used as a detector. Polarization resolved measurements were obtained using a 5 m length of polarizing optical fiber (providing a polarization resolving ration of ~30 dB) attached to the end of the corresponding reading port (only CH1 or CH2) and connected directly to the OSA, while being supported in a rotating v-groove for being aligned with the coordination system of the microsphere.

## 3. Results and Discussion

**Figure 4:**
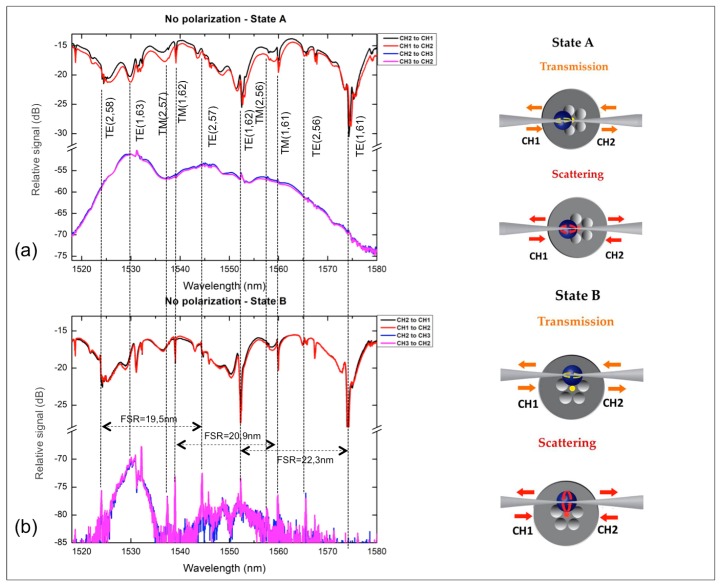
Both states A and B for transmission and scattering are measured and plotted at first with an SMF28 without using polarizing optical fiber. The coupling between the SOFT and the core of the MOF taper in State A (**a**) is obvious by observing the elimination of clear and sharp resonances whereas the geometry of State B (**b**) allows us to obtain more significant resonances in the scattering spectra.

Transmission spectra of the T-junction for different polarization states, port excitation and reading, for State A and State B are presented in Figure 4.

The transmission spectra of the SOFT in contact with the BaTiO_3_ microsphere show WGMs behaviour (Figure 4, CH1 to CH2 and vice versa) and are typically obtained for microspherical cavities. The azimuthal *l* and radial *q* modal orders (TE, TM(*q*, *l*)) have been estimated assuming a nominal BaTiO_3_ microsphere diameter 25 µm (as measured using SEM scans) and a refractive index of 1.9 [3]. A particular feature of the WGM spectra of the State A is a modal distortion by means of broadening observed for particular WGM orders; this is related to the perturbation resulted from the wedging of the BaTiO_3_ miscosphere into the MOF capillary end face. Typical Q-factors for the spectra of State A of Figure 4 are ~700, well below values reported for BaTiO_3_ microspheres of similar diameter and optical fiber taper excitation [23]; this is related to the great mismatch between the effective indices of the WGMs and both the MOF and SOFT [24]. Accordingly, the resonant wavelengths of WGMs excited with the SOFT and scattered from the microsphere were collected and measured with the MOF (CH2 to CH3, State A), proving the possibility of using the T-shaped system for the light routing. Yet, this spectra (CH3 to CH2 and vice versa-State A) are characterized by a strong background continuous signal emerging from the light coupling from the BaTiO_3_ microsphere to the two waveguide systems, however not through the WGM resonation, but from a standard total internal reflection cancellation and a standard lensing process. This strong and broad background can also shadow WGM mediated coupling from the MOF to the SOFT.

The spectral data for State B excitation presented in Figure 4 exhibit specific similarities with those corresponding to State A. Several of the WGM notches for both polarizations spectrally coincide with those of State A, with relative spectral shifts between the two States that are mostly attributed to possible eccentricity of the capillary wedged BaTiO_3_ microsphere. A point of particular interest is related to the Q-factor of the WGM excited for State B, which appears much greater compared to the one obtained for the same microsphere, while being positioned and excited at State A. For State B the Q factor was calculated to be 1.63 × 10^3^ for the scattering spectra whereas for State A it was calculated to be ~500 [4,15]. This may be attributed to the fact that for State B the positioning of the wedged microsphere with respect to the MOF symmetry is different, with the MOF core not being aligned with the SOFT axis. This affects the excitation conditions of the systems both through the SOFT and the MOF [25,26]. The WGM peaks measured for light routing between the MOF and the SOFT (CH2 to CH3 and vice versa), exhibit a higher signal to noise ratio with respect to those of State A, since the background signal is lowered by almost 30 dB (at wavelength 1530 nm). This significant reduction of the background, broadband signal denotes that light coupling is minimized through lensing effects and total internal reflection cancellation, but rather takes place through the WGMs resonation facilitated at the BaTiO_3_ microsphere.

An interesting point for the State B light routing scheme is that while the MOF core and the SOFT axis do not coincide/align in space, light coupling between the two vertically placed waveguide structures still exist through WGM resonations with polar modal numbers |*m*| ≠ *l*, where *l* refers to the azimuthal WGM order [27]. In Figure 4 we denote resonances for the two first radial orders along with the azimuthal number for TE and TM modes [4,10]. The free spectral range (FSR) measured in the graphs is in good agreement with the theoretically expected values based on the FSR formula and the corresponding resonant wavelength [1]. In such a light coupling scheme WGMs with *|m| ≠ l* will be delocalized from the azimuthal circumference of the BaTiO_3_ microsphere defined by the excitation plane of the SOFT [28], possibly allowing modal crossing between modes of high difference between *m* and *l* modal order, facilitated by the microsphere wedging perturbation and eccentricity of the microsphere.

**Figure 5:**
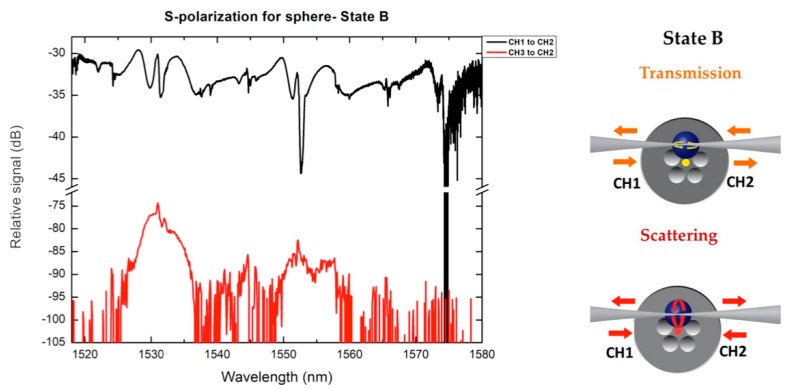
State B: Transmission and corresponding scattering spectra of the T-shape system obtained with the polarizing optical fiber.

In another set of measurements, we used the polarization maintaining optical fiber for both States A and B for resolving the two polarization states. In State A because of the position of the sphere with respect to the SOFT, the fiber s-polarization excites mostly TM modes for the microspherical resonator, whereas in State B the fiber s-polarization corresponds mostly to TE modes for the resonator (‘slapping’ and ‘piercing’ polarization). The majority of polarization resolved data obtained appeared quite noisy, hindering WGM resonation features especially in the SOFT to MOF coupling. In general, due to the geometry of the specific WGM resonation scheme, we expect polarization cross-coupling to take place [29], decrementing polarization resolved modal measurements as shown in Figure 5.

## 4. Conclusions

We communicate results on the investigation of light coupling and routing via a microspherical resonator integrated in a T-shaped optical fiber configuration system, constituted of a BaTiO_3_ microsphere wedged in the capillary of a grapefruit shaped optical fiber while being excited for different orientations using a single mode optical fiber taper. The specific optical configuration was spectrally characterized in a multi-portal arrangement and the light routing results were discussed in conjunction with basic WGMs formulation, showing the possibility to rout the light in a 90° angle system from the SOFT to the MOF and vice versa. We intend to continue our investigations of the specific light coupling and routing system for potential use in self-feedback systems for active glass microspherical cavities, where the MOF end will be spliced to one end of the SOFT. Another possible application refers to optical routers and microspherical lasers arranged in three dimensions [6], while being pumped through a single optical fiber taper with specific spectral signatures per MOF port [30]. Preliminary results in tuning the coupling between the SOFT and the MOF through the WGM spectral comb by exploiting the photorefractivity of BaTiO_3_ microsphere, have resulted in typical spectral shifts of the WGMs by ~0.3 nm for a exposure dose of 10.8 J, using a 405 nm CW solid state laser.
